# 

**Published:** 1905-04-29

**Authors:** 


### NOTICE TO CORRESPONDENTS.

All MSS., Letters, Books for Review, and other matters intended for the Editor should be addressed The Editor, "The Hospital," The Hospital Buildings, 28 & 29 Southampton Street, Strand, W.C.

The Editor cannot undertake to return rejected MS., even when accompanied by stamped directed envelope.

### Notice to Advertisers and Subscribers.

All Advertisements, Orders for Copies of The Hospital, and Business Communications should be addressed to **THE MANAGER (Not to the Editor),** The Hospital, 28 & 29 Southampton Street, Strand, London, W.C.

The Cost of Subscription, post free, is at follows:—Per week, 3½d.; per quarter, 3s. 3d.; per half-year, 6s. 6d.; per annum, 13s. Foreign and Colonial:—Per annum, 19s. 6d., payable, in all cases, in advance.

**NOTICE.—Vol. XXXVII. of "The Hospital" October 1904 to March 1905), in handsome cloth binding, will shortly be on sale at the office of this Journal, price 10s. 6d. Cases for binding the Numbers contained in this Volume 2s. Index to Vol. XXXVII., 3½d., post free.**

**The Monthly Part for April is now ready. Price 1s., by post, 1s. 3d.**

### BOOKS RECEIVED.

Baillière, Tindall and Co.

"The Principles and Practice of Asepsis." By A. S. Vallack, M.B.

John Bale, Sons, and Danielsson.

"What Venereal Diseases Mean, and How to Prevent Them." By Professor Erik Pontoppidan.

John Dicks.

"Dicks' London Street Guide."

J. Wright and Co., Bristol.

"Errors of Refraction." By Chas. Blair, M.D.

W. H. and L. Collingridge.

"The City of London Directory, 1905."

Elliot Stock.

"The History of the Society of Apothecaries of London." By C. R. B. Barrett, M.A.

Spottiswoode and Co.

"The Dentist's Register, 1905."

A. Owen and Co.

"Alcoholic Poisoning and Degeneration." By Professor G. Bunge, M.D.

J. and A. Churchill.

"The Book of Prescriptions." By E. W. Lucas, F.I.C.

### Medical Appointments.

**George Ashton**, M.D.Manchester, M.R.C.S.Eng., L.R.C.P. Lond., reappointed Assistant Surgical Officer to the Manchester Royal Infirmary. **F. A. Bainbridge**, M.D., D.Sc., M.R.C.P., Gordon Lecturer in Experimental Pathology, Guy's Hospital. **L. A. Clutterbuck**, M.B., B.S., Clinical Assistant to the Chelsea Hospital for Women. **A. J. Coddon-Fletcher**, M.R.C.S.Eng., District Medical Officer of the Melton Mowbray Union. **Arthur Bernard Cridland**, F.R.C.S.Edin., M.R.C.S.Eng., L.R.C.P.Lond., Honorary Ophthalmic Surgeon to the Staffordshire General Infirmary. **Sydney W. Curl**, M.A., M.D.Cantab., M.R.C.P.Lond., Honorary Physician to the Essex and Colchester Hospital. **John Howell Evans**, M.Ch., F.R.C.S., etc., Assistant Surgeon to the Cancer Hospital. **J. Dudgeon Giles**, M.B., B.S.Edin., House Surgeon to the North Riding Infirmary, Middlesbrough. **H. B. Gladstone**, M.D.Edin., District Medical Officer of the Lewisham Union. **J. R. Higson**, M.B., C.M.Edin., D.P.H.Camb., Certifying Factory Surgeon for the Lewisham District. **R. Hughes** M.B.Lond., M.R.C.S., Medical Officer of Health, Fenton Urban District. **A. W. D. Hunt**, M.R.C.S., L.R.C.P.Lond., Certifying Factory Surgeon for the Chagford District, Devonshire. **A. Hutley**, M.B., C.M., Senior House Surgeon to the West Bromwich District Hospital. **A. J. Jackson**, L.R.C.P., L.R.C.S., Edin., Second Assistant Medical Officer to the St. Marylebone Infirmary. **J. Kenrick Jones**, L.R.C.P.&S., L.F.P.S.Glasg., Medical Officer of Health, Llansilin Rural District Council. **Charles R. Keyser**, F.R.C.S.Eng., Assistant Surgeon to the Cancer Hospital, Fulham Road, S.W. **F. C. R. M. Knight**, M.R.C.S., L.R.C.P.Lond., District Medical Officer of the Holborn Union. **E. Browning Lathbury**, M.R.C.S., L.R.C.P.Lond., House Surgeon to the Herefordshire General Hospital. **Hugh Lett**, M.B., Ch.B.Vict., F.R.C.S., Assistant Surgeon to the London Hospital. **F. S. Mackenzie**, M.B., Ch.B.Edin., House Surgeon to the County Hospital, York. **H. S. Marsh**, M.B., First Senior Resident Medical Officer, Sydney Hospital, N.S.W. **A. A. McLarty**, M.D., (U.S.A.), Clinical Assistant to the Chelsea Hospital for Women. **R. T. Meadows**, M.D.Edin., D.P.H., Medical Officer of Health St. Germans Rural District. **Alex. Mitchell**, M.B., Ch.B., Assistant House Surgeon to the West Bromwich District Hospital. **T. H. P. Morris**, M.R.C.S., L.R.C.P., Medical Officer of Health, Newcastle-under-Lyme Rural District. **E. F. Page**, M.B., B.S.Birm., District and Workhouse Medical Officer of the Solihull Union. **G. A. Phillips**, M.R.C.S.Eng., L.S.A., Certifying Factory Surgeon for the Walsall District, Staffordshire. **R. H. J. Swan**, M.S., F.R.C.S., etc., Surgical Registrar to the Cancer Hospital. **William Thompson**, M.B., Ch.B.Vict., Medical Officer of Health, Mytholmroyd Urban District.

A SELECTION OF

### Nursing Manuals

*Published by*

THE SCIENTIFIC PRESS, Ltd.,

28 & 29 Southampton St.,

Strand, London, W.C.

**AT 3/6**

**NURSING: ITS THEORY AND PRACTICE.**
By Dr. Percy G. Lewis.

**SURGICAL WARD-WORK AND NURSING.** With particulars of Instruments used in various operations. By Alexander Miles, M.D. **3/10** post free.

**OPHTHALMIC NURSING.**
By Sydney Stephenson, M.B. **3/10** post free.

**DIET IN SICKNESS AND IN HEALTH.**
By Mrs. Ernest Hart.

**OUTLINES OF INSANITY.**
By Francis H. Walmesley, M.D. Popular Edition **1/6.**

**FIRST AID TO THE INJURED AND MANAGEMENT OF THE SICK.** By E. J. Lawless, M.D.

**AT 2/6**

**ELEMENTARY ANATOMY & SURGERY FOR NURSES.**
By Wm. McAdam Eccles, M.S., M B.

**HOSPITAL SISTERS AND THEIR DUTIES.**
By Eva C. E. Lückes.

**NURSING IN DISEASES OF THE THROAT, NOSE, AND EAR.** By P. Macleod Yearsley, F.R.C.S.

**MEDICAL GYMNASTICS: Including the Schott (Nauheim) Movements.** By Axel V. Grafstrom, M.D.

**SPINAL CURVATURE AND AWKWARD DEPORTMENT.**
By S. G. Muller. English Edition by Richard Greene, F.R.C.P.

**AT 1/=**

**HOSPITAL AND PRIVATE NURSING.**
By Miss S. E. Orme.

**FEVERS AND INFECTIOUS DISEASES.**
By Wm. Harding, M.D.

**MIDWIVES' POCKET BOOK.**
By Honnor Morten.

**GYNÆCOLOGICAL NURSING.**
By Hawkins Ambler, F.R.C.S.

**NOTES ON PHARMACY & DISPENSING FOR NURSES.**

By C. J. S. Thomson.

**THE MODERN NURSING OF CONSUMPTION.**
By Dr. Jane Walker. **1/2** post free.

**THE EXAMINATION OF THE URINE.**
By Dr. J. K. Watson. **1/1** post free.

**AT 6d.**

**ON PREPARATION FOR OPERATION IN PRIVATE HOUSES.** By Stanmore Bishop, F R.C.S.

**MODERN NURSING IN PRIVATE PRACTICE.**
By Sir William Bennett. **7d.** post free.

**SCHOTT TREATMENT FOR CHRONIC HEART DISEASES.**
By Richard Greene, F.R.C.P.

**POCKET BOOK OF TEMPERATURE CHARTS.**
Containing 17 Twelve-Hour and 8 Four-Hour Charts. **7d.** post free.

**"SIMPLEX" DISTRICT NURSES' CASE-BOOK.**
Containing space for 50 cases, ruled and printed. **7d.** post free.

NEW CATALOGUE OF NURSING MANUALS POST FREE ON APPLICATION.

**THE SCIENTIFIC PRESS, LTD.,** Publishers and Booksellers of all kinds of Nursing Text-books, Case-books, Charts, &c.

**28 & 29 Southampton St., Strand, London, W.C.**

